# Discussing human values in digital immortality: towards a value-oriented perspective

**DOI:** 10.1186/s13173-021-00121-x

**Published:** 2021-11-26

**Authors:** Vinícius Ferreira Galvão, Cristiano Maciel, Roberto Pereira, Isabela Gasparini, José Viterbo, Ana Cristina Bicharra Garcia

**Affiliations:** 1 Institute of Computing, Federal University of Mato Grosso (UFMT), Cuiabá, Brazil; 2 Department of Informatics, Federal University of Paraná (UFPR), Curitiba, Brazil; 3 Institute of Computing, Santa Catarina State University (UDESC), Joinville, Brazil; 4 Institute of Computing, Fluminense Federal University (UFF), Niterói, Brazil; 5 Department of Aplied Informatics, Federal University of the State of Rio de Janeiro (UNIRIO), Rio de Janeiro, Brazil

**Keywords:** Digital legacy, Digital immortality, Human values, Digital memorial

## Abstract

Intense social media interaction, wearable devices, mobile applications, and pervasive use of sensors have created a personal information ecosystem for gathering traces of individual behavior. These traces are the digital legacy individuals build all through their lives. Advances in artificial intelligence have fed our dream to build artificial agents trained with these digital traces to behave similarly to a deceased person, and individuals are facing the possibility of immortalizing their ideas, reasoning and behavior. Are people prepared for that? Are people willing to do that? How do people perceive the possibility of letting digital avatars take care of their digital legacy? This paper sheds light on these questions by discussing users’ perceptions towards digital immortality in a focus group analysis with 8 participants. Our findings suggest some key human values must be addressed. These findings can serve as preliminary thoughts to inform system design, from the very early stage of development, that preserve the digital legacy while respecting the human needs and values concerning the delicate emotional moment that death provides. This qualitative research analyzes the data, and based on the insights learned, proposes important considerations for future developments in this area.

## Introduction

Information technology has occupied an important role in our daily lives as a tool to deal with the constant information avalanche we are exposed to. We have been adapting to this evolving reality that makes us dependent on technology. The changes of our way of life due to technology imply a reorientation of the knowledge and interests that guide our behaviors [1]. These transformations bring topics that challenge our perception about the role of technology in our own taboos, one of which is death. Sellen et al. [2], discussing the human values in the digital era, claim the “end of the ephemeral” as one of the major changes in the way we relate to technology.

The constant exposure of our lives on social media leaves our personality traits and personal data available forever on the net and at easy reach without our consent. We may leave, but our digital self remains [3]. Death is a human experience that is emotionally salient, contextual, and culturally dependent. Therefore, the concept of digital immortality and the application of it through digital legacies, chatbots, and other kinds of digital technologies will also necessarily be highly nuanced and potentially contentious.

Existing computational techniques, such as machine learning, with the social net users’ digital traces allow computers to simulate some aspects of the deceased users’ behavior, immortalizing their digital presence [4].

A key issue between digital legacy and digital immortality is the possibility of posthumous interaction^1^ with the frozen user’s profile.

There are legal and ethics issues such as [5–7]: Who owns the data related to the deceased if he/she has not delegated an heir? If a perfect digital copy of the deceased is possible, should it be treated similarly as any human being? Is digital immortality a relief or a hindrance in order to help people to deal with grief? Who is responsible for a bad behavior from a digital copy of the deceased? Should the deceased digital copy be allowed to evolve by interacting with other? These are important questions that this paper started to address. We believe our findings bring evidences and shed some light on the importance of further investigation.

According to Bell and Gray [8], there are different types of immortality, such as one-way immortality, wherein data from the deceased can be exposed and preserved for future generations, such as in digital memorials and two-way immortality, wherein via digital artifacts, people not only have access to the deceased data but can actually communicate and interact with an artificial version of the deceased. These artificial profiles, created from the deceased data, can learn and evolve from interacting with the world. Chatbots and avatars that replicate the deceased users are good examples of using technology to accomplish this level of immortality.

The possibility of interacting with artificial profiles created from the deceased data raises natural issues and concerns through achieving digital immortality. Ohman [9] points out the growing interest of using digital immortality for commercial purposes.

In science fiction movies and TV series, such as, Ghost in the Shell, Altered Carbon [10], Metropolis [11], Westworld [12], and the Black Mirror episode: “Be Right Back” [13], the role of humanity—including what it means to be a human being and what our human values are in the face of technological progress—are frequent discussion topics. “Be Right Back,” in particular, was the inspiration for the research we present in this paper. In this Black Mirror episode, a person created an artificial version of a deceased loved one, as a type of “resurrection,” to deal with the pain of death. Initially, immortality manifests itself in software and, gradually, via hardware, leading to a series of moral, ethical, social, and personal issues on the subject.

The memory of the deceased may be disturbed with a post-mortem relation with a digital copy of him/her [14, 15]. Among the questions raised in the episode is the issue: “What human values related to technology are involved in digital immortality?” This question is the focus of our study. Human values as discussed by Andrade, Bispo, and Dos Santos [16] do possess a wide range of definitions, be the moral and spiritual fundamentals of the human consciousness, the concept that gives objectivity to their actions in a moral or metaphysical capacity, or even the inherent human characteristics that guide societal well-being. In this paper we will be mainly considering the human values prospected by Pereira and Baranauskas [17], which are presented by them as primarily predominant in social software design.

The current *COVID-19* pandemic has also highlighted the need for more platforms to properly pay homage to our dead in a more active manner, such as digital graveyards [18, 19], or to better prepare our legacy for the next generation with the aid of end-of-life decisions support systems [20].

The question of immortality brings a set of multi perspective doubts without easy answers. Carreira [21] raises some important questions that should be answered before jumping into technological missions, such as: Will immortality be extended to everyone? Who will trigger the memories? Where will be the human: in the living being or in the memory? These questions demand answers and debate before immortality becomes technically feasible. In an investigation with users on this topic, Galvão et al. [7] highlight the idea that allowing oneself to exist forever in the digital world will be less important than understanding the positive and negative effects on people’s lives with such a technology. We must not, at any stage, neglect the human needs that make these ideas possible.

There has been a growing interest in the possibilities of creating digital immortality, either discussing the technological aspects [1, 8, 22], the philosophical issues [23], the legal implications [24, 25], the economic impact [9], the psychological changes on people [26], and the physical aspects of it [27]. However, during our literature review, we noticed that immortality has been little discussed from the perspective of users as the interested parties affected by this technology. This study focuses on understanding the expectations of users from systems that seek to immortalize and keep the memories of the loved ones alive in an interactive format. For this investigation, we conducted a series of focus group interviews to obtain the opinions of volunteers regarding technologies that promise digital immortality through digital memorials, software or hardware. According to the volunteers’ opinions and the discussions emerged during the interviews, this paper presents an analysis based on the human values mapped on social software by Pereira and Baranauskas [17] responding to the focus of this study and offering a starting point for a value-oriented reflection on digital immortality.

This paper^2^ is organized as follows. The “Digital immortality” section brings a deeper discussion about this problem domain. The “Design values and value-sensitive design” section presents the main concepts about such research topic. The “Existential HCI” section discusses the existential nature of these computational tools. The “Research methodology” section details the conduction of the focus group interviews and the qualitative analysis of the transcribed interviews. The “Findings” section presents a taxonomy of the values that study participants were most concerned when talking about digital immortality. Finally, the ”Final considerations” section presents the contributions, limitations, and future work for this research.

## Digital immortality

A legacy, according to Crocker and McLeod [29], could be anything, such as, documents, music, photos, playlists, visualization history, social network profiles, and even the hardware for storage, that are left behind to last, as a message of sorts to people of the future. Aside from the environment, digital and physical legacies are very similar in purpose.

The means to detect and delegate destinations to a digital legacy is still a major challenge in human-computer interaction (HCI) in Brazil [30] and in other countries as well. As more people pass away, more digital legacies are lost or inaccessible to families and loved ones, either by unfamiliarity or disinterest.

In the present, companies like Facebook and Google offer options to users to destine their legacies, after confirmation of their deaths, to an heir or even complete transformation of their digital accounts into digital memorials [5]. Digital services like Safebeyond and Miigen also offer specialized treatment of posthumous data. The treatment of a digital legacy is not yet widely advertised or implemented in most software and hardware. The fact that laws and policies are slow to catch up with technological progress means that the proper legal resources to solve human issues that accompany such advancements are essentially in a gray area, and tech companies have to self-regulate as best as they can. In general, there is legislation around the world that allows legitimate heritage of digital data [24, 31–33]; however, the inheritance process is not as comprehensive as a material legacy inheritance.

Alongside all the previously mentioned alternatives for the digital legacy, an alternative that has been slowly earning interest is digital immortality. The reason for such an interest is for allowing the living the opportunity to interact with the data of the dead under many different forms, effectively re-contextualizing the meaning of death and its manifestations in the digital space.

One of the many definitions associated with immortality is provided by the Encyclopedia Britannica [34]: “The term *immortal* has been used in a wide general sense for everlasting, as the writings of Plato, the plays of Shakespeare, the music of Mozart are immortals. But in its chief use the term *immortality* has referenced a continuity of people’s spiritual existence after the death of their bodies.”

For philosophers like Plato [35], immortality is given through something that all living beings have, a soul, which contains all the knowledge we have before we have a body. According to the Oxford Dictionary of Philosophy [36]: “In Platonic and Neoplatonic traditions, immortality may be given a ‘timeless’ twist. Instead of living forever the goal is to live altogether (...) the foregoing points apply to personal immortality, or at least the continuous existence, of some of a person’s features may well survive death. One’s work, or fame, or notoriety, or genes may well survive in the minds and bodies of others.”

Similarly, digital immortality refers to the preservation of a user’s digital identity, keeping it active even after the user’s death [8]. Digital immortality can be planned or simply occur as a result of the effort of others to keep one’s memory active.

**Fig. 1:**
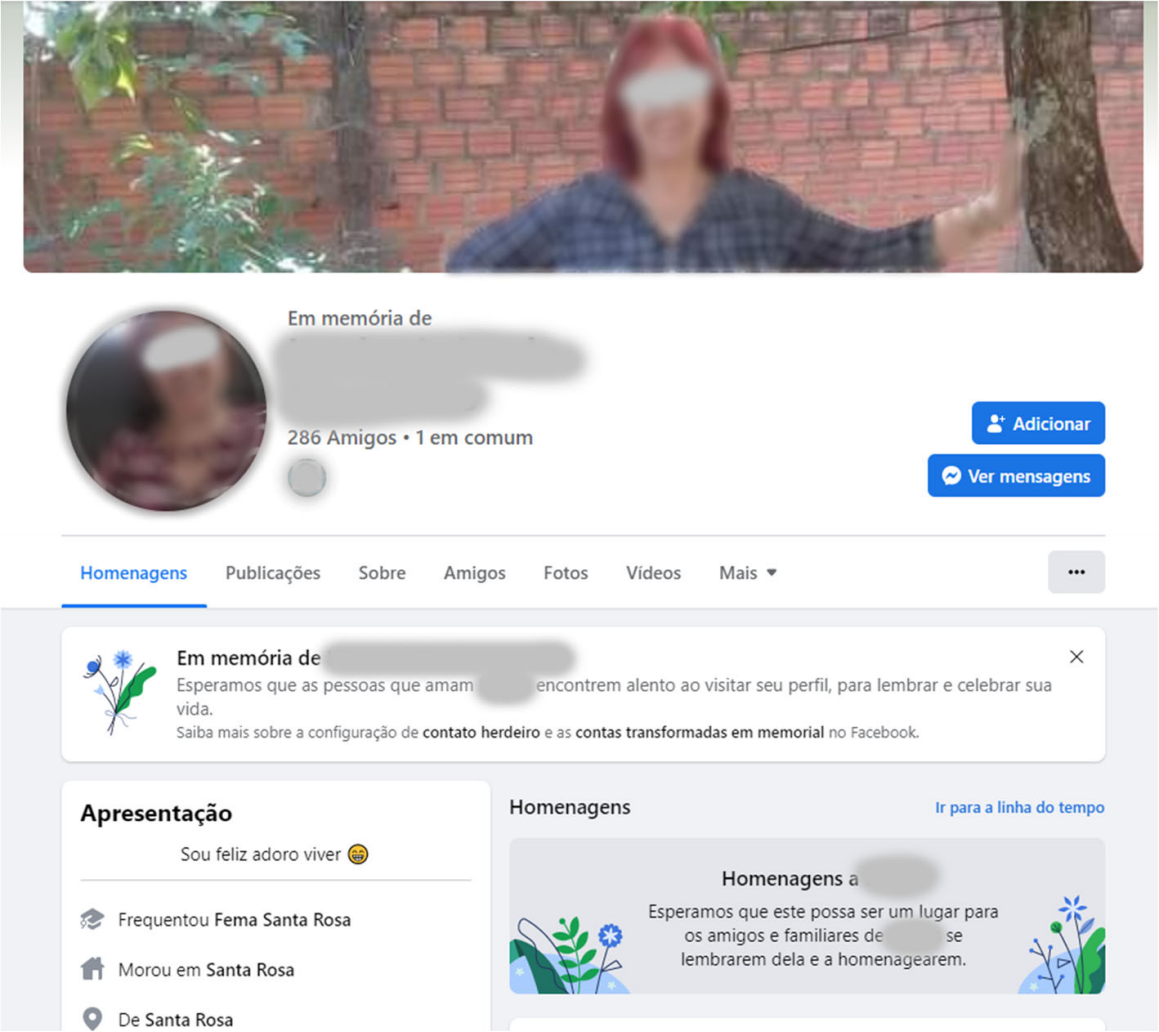
Example of a digital memorial on Facebook
- Creating digital agents able to behave as the deceased, which are trained with the deceased message patterns. The tuned intelligent chatbot would be able to send textual messages similar to those the deceased sent when alive (immortalizing the ideas using software), as illustrated in Fig. 2; and

For Galvão et al. [37], there are three options for immortalizing a user:

- Setting up a digital memorial, which keeps online records of the deceased users, generated in life or in the form of posthumous tributes (immortalizing itself in a software), as illustrated in Fig. 1;

**Fig. 2:**
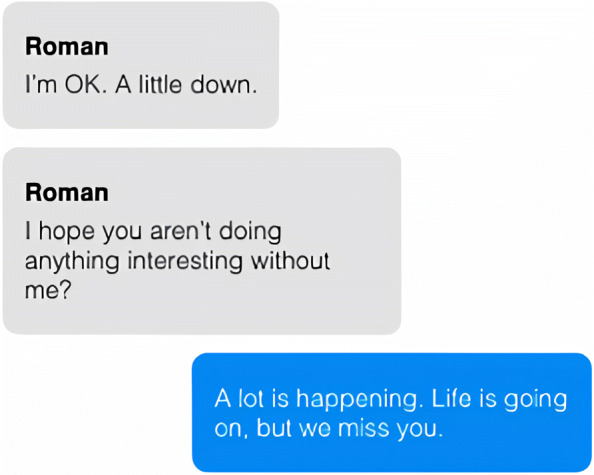
Example of a chatbot customized to performed as a person [38, 39]
- Transferring the memories of the deceased users to a mechanical body and, thus, immortalizing them through their artificial body—known as “avatar,” with data transferred via software (immortalization in hardware), as shown in Fig. 3.

**Fig. 3:**
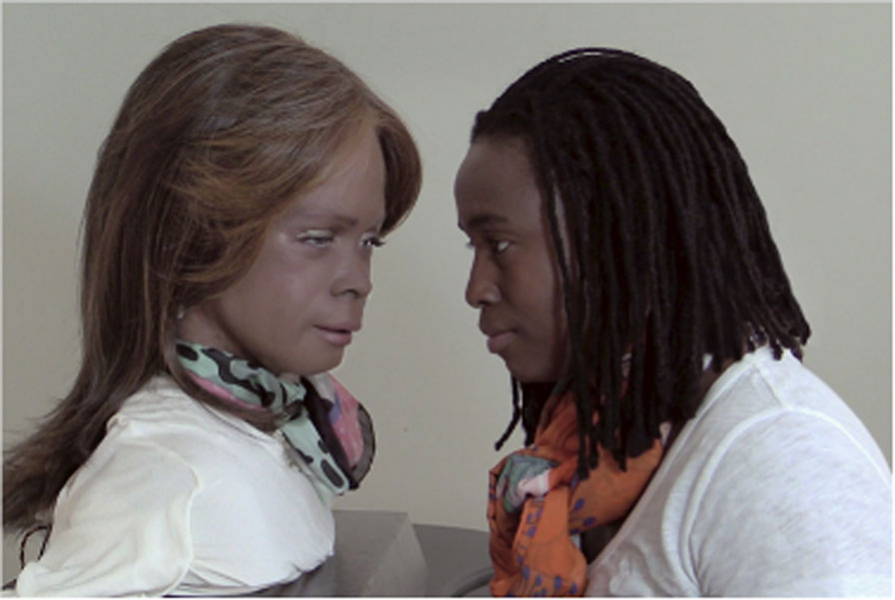
Reporter and BINA-48 robot conversation [40]

Any information left by people on the Web is part of their digital legacy immortalized on the Internet. Users keep data dispersed on the Web in many applications, with or without logged access. Thus, the immortalization of users in software, through their data, is a reality. In social networks like Facebook, the transformation of a user’s profile into a digital memorial, or its exclusion, after the user’s death, is one of the features implemented and already available [41]. When the death of a user is proven or automatically detected, the system performs what was configured by the user in the terms of use if the deceased user has configured that in the system. The transformation in digital memorial is the default Facebook option.

For Crocker and McLeod [29], the *memorialization* of social media profiles and the growth of memorial websites allow people to continue to exist in the daily lives of friends and family. For the authors, this phenomenon is relatively new in West’s postindustrial era.

Naturally, given the possibility of immortalizing a person and allowing posthumous interactions with their data, system developers are faced with complex issues, both philosophical, technical, and social, with those predicted by Galvão et al. [28]:

- When manipulating a person’s data, one can be changing the “digital self,” that is, his digital identity. Is it okay to do that?
- Should these digital immortals be treated like a human being? Is there a need for special treatment for them?
- Do machines only display the intelligence of their programmers or is it their own independent intelligence?

Such questions arise from something that normally does not receive due attention in the creation of computing systems: human values. As a continuation of the studies by Galvão et al. [42], there are some complex issues concerning digital immortality: the differences when dealing with famous deceased versus non-famous deceased individuals, explicitly defined public data versus private data, rights and duties related to data preservation versus deletion, technological advances versus cultural traditions, and the reasons for desiring and justifying the use of digital immortalization.

## Design values and value-sensitive design

While technical software/hardware issues can be dealt with relatively ease by experts and professionals in the field, social and organizational issues are still challenging. There are not always simple solutions about the role and use of technology, especially when dealing with controversial issues such as death. Value-sensitive design (VSD) is a methodological framework developed by Friedman, Khan, and Borning, focusing on addressing the design of technologies so that they prioritize and respect human values [43, 44]. This framework is particularly interesting for HCI as it supports conceptual, empirical, and technical investigation to find out the socio-technical problems of emerging technologies [45].

The VSD approach has as key features [45]:

1. Affect the design of a technology from its conceptualization to its development;
2. The understanding of values in this framework is not limited to just one type, professional, social, ethical, cultural, political, economic, educational, entertainment, and other similar items that can be considered;
3. Expands the concepts of participatory project (participation, democracy and cooperation),to include all values relevant to the context in which they are being applied;
4. Contributes to the design process through its conceptual, empirical and technical approach;
5. Technological solutions must be developed respecting the values supported by an individual or group.

According to Friedman, there are three important domains with regard to human values: moral, conventional, and personal [44]. For the authors, the moral domain is related to judgments that people do based on notions of justice, equality, or human well-being. In contrast, the conventional domain is related to uniformity or patterns of behaviors developed to make social interactions work, and the personal domain is related to issues that belong to an individual’s jurisdiction.

The importance of values in interactive systems design goes beyond simple convention. Pereira and Baranauskas [17] highlight that aspects related to culture, such as values, beliefs, and behavioral patterns, influence the way technology is understood, adopted, and used, as well as the impact it may cause on the environment and on people. As an example of the influence that values have on software design, Pereira et al. mention the functioning of ChatRoulette [17], an application that was designed to randomly connect people in audio and video conversations. For the authors, the system appeared to favor ephemeral interactions as it did not require any information that could identify users or that could compromises their privacy, since users were in control of the content they were transmitting (image, sound and text). Because people *rolling the roulette* would stopping watching someone in order to watch a different user, there were minimal concerns of participants feeling rejected since there was no clear system intention of supporting social ties via continuous interaction. Instead, the instantaneous judgment promoted creative behavior as users vied to keep the attention of others.

**Fig. 4:**
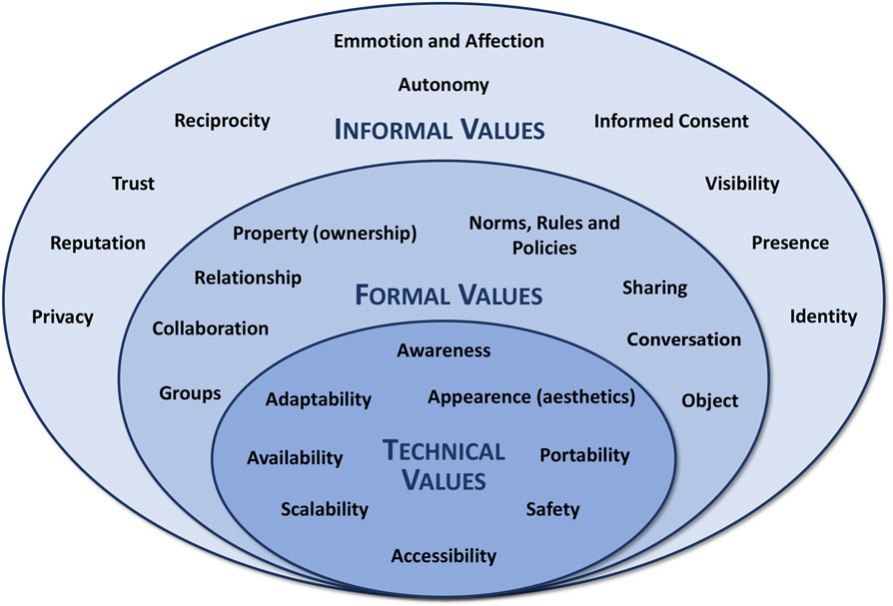
Personal values (informal in the onion border layer), social values (formal in the middle layer), and technical (as the inner layer of the onion), based on Pereira and Baranauskas [47]

Pereira and Baranauskas [17] propose a classification for values according to their informal, formal, and technical nature based on the Organizational Onion artifact. Proposed by Ronald Stamper [46], the artifact represents these three levels in a structured form: the informal, where organizational culture, customs, and values are reflected as the beliefs, habits, and patterns of individual behavior of people; formal, in which rules and procedures are created to replace meanings and intentions; and the technical, representing the computing system located within the formal level. Figure 4 presents a set of values identified in the context of social software and classified according to the Semiotic Onion levels [47].

These values, despite being in different sections of the onion, are intrinsically linked and are based on human aspects present in societies and legislation. For example, the value of Privacy is defined by Pereira and Baranauskas [47] as: “the requirement or right of an individual to determine what information about him can be exposed and who can access it.” When a message is sent for someone, whether by physical or electronic means, it is expected that it will arrive in full form to its addressee: something that is present in Article 5, Item X of the Brazilian legislation of 1988 [48]. When addressing the informal value, Privacy implies the involvement of formal values such as Norms, Policies and Rules (the formal rules that govern a system); Sharing (the possibility to make information available to other individuals); and technical values such as Security (if the information is protected) and Usability (whether the interface and use of the systems are practical and satisfactory). When working with values in the development of systems, one value will always relate to others.

While Friedman’s VSD [44] provides guidance on how to approach values, Pereira and Baranauskas [47] offer additional support on what is important to consider when approaching values—notably, they introduce the technical dimension and draw attention to the relation between values. Therefore, both approaches can work together and be combined to inform design activities.

For this particular study, the VSD framework was chosen due to its human centric approach. By combining both VSD and the social software values, we sought to explore the human issues regarding digital immortality—VSD also provides a wide and interdisciplinary point of view, which is appropriate for the many complex questions surrounding our study focus. Research by Pereira et al. [17] on human values are focused on social software. Whether intentional or not, software that promotes digital immortality will also influence values and behaviors in its users by design. Identifying some of the key user’s concerns is a necessary first step in order to develop solutions that cater to these needs.

As Balkin points out [49]: “Human beings use culture for at least three interlocking and interrelated purposes. The first is to get about the world, to understand it and make use of it. The second is to interact with others as others, rather than as objects of manipulation. The third is to articulate and express human values.” Consequently, the concept of human values can be extended, reaching digital legacy and its impact on society and its different cultural values. The issues of human values and the digital legacy are among the great challenges of human-computer interaction and information systems in Brazil [50, 51], demonstrating the recognition of these communities about the importance and the need to advance in the understanding and consideration of values when working with interactive computer systems.

While digital immortality is not widely used or formally recognized as a social software, the values and approaches taken by the previously mentioned authors are considered in this study in order to understand what are users’ key concerns about its usage, how it may be presented, how it operates, etc. Having a better grasp of what users find interesting or problematic about digital immortality is an important step in order to guide future technologies in grasping its limitations for immortality, what users expect, and what needs to be carefully approached before, during and after development of solutions that deal with such a sensitive and mostly unknown topic. If careful examination of these is not properly done, digital legacies can become inaccessible, people can have their digital goods harmed, and it could create tech that users do not find approachable, useful, respectful, etc. These effects also can affect by proxy digital immortality and the means to achieve it. Technologies reflect our values, but also influence our values.

## Existential HCI

Associated with issues such as design values and digital immortality, another important factor is the existential nature of these tools. Kaptelinin [52] argues that according to the evolution of technology, HCI must also cover issues of existential nature (what the author calls the existential HCI). This particular perspective focuses on addressing broad life issues such as “what is the meaning of life?”, “who am I really?”, and “what will happen when I die”. Kaptelinin reinforces that this approach is necessary, especially when investigating the moral and ethical aspects of technologies designed for sensitive issues.

While the impacts of AI technology for digital immortality are still largely unknown, through the existential IHC, Kaptelinin [52] recommends the prioritization of the following aspects of study:

1. The focus on existence in general, instead of pragmatic tasks;
2. The search to understand how people confront their own mortality;
3. The need for space for existential decisions (culture, creation, religion, personality and relationships and among other personal factors of an individual affect his or Her decisions during life);
4. The unpredictability of existential experiences.

According to the author, by taking these factors into account, it is possible to recognize and understand what intimately human experiences can be treated appropriately through technology.

As Balkin argued [49], human values are deeply linked to the human experience as a whole, in every aspect, from more objective and collective matters to the more introspective and abstract ones. It is only natural to infer that these values are brought from the very nature of human existence, that is to say, an existence filled questions which none of them have an easy/comfortable answer [53]. No matter which human work, human values are consciously and unconsciously printed in them. This study is no exception. The existential HCI perspective in the context of this research is helpful to understand what are participants’ opinions about digital immortality, both superficially as well as well as more subtler and subdued implications behind their words, and how these implications are categorized from a VSD perspective.

Last, but not least, digital immortality brings the challenge of protecting the deceased’s data integrity from hackers [53].

## Research methodology

In order to investigate the issue of digital immortality and the human values involved in technologies designed for this purpose, the focus group methodology was chosen for this study [54].

The methodology allows participants, through the discussion of the theme, to build knowledge in a participatory and active way. And for researchers, it gives an opportunity to observe the evolution of the participants’ opinions and to perform a qualitative study structured with the aid of materials (such as software, videos or physical items), enabling the participants to carry out the discussions in a constructive way, in a relatively short period of time. The focus group methodology was chosen since it allowed for observation of participants confronting their opinions in favor or against the topic. By uniting people with different expertise, we can observe aspects or concerns that were not initially considered (e.g., a psychologist can point out the issues digital immortality may have on the families of the deceased), as well as observe what is more or less interesting about the topic to a diverse subset of participants across different age ranges, genders, and life experience.

Through the investigation of the main concerns of users, this method can point out suggestions for the design of systems that promote digital immortality and the digital legacy supporting a better understanding of human needs. Based on Friedman’s suggestions [55] for considering conceptual and methodological issues, the study of this article was guided by the questions of Galvão et. al. [28] and in conjunction with the Black Mirror episode “Be Right Back” [13]. Such methodology involves intense discussion and interviewing of a small group of people, with a given focus or question [56] and opens various avenues for discussions and perspectives not previously considered by the authors.

In this study, the focus and purpose of the group was to understand how users perceive the issue of digital immortality. The process of preparing and holding this group meeting lasted 2 weeks, consisting of:

- Creation of a semi-structured script interview;
- Preparation of terms of consent to use users’ images, audio, and text for research previously approved by the university’s ethics committee,^3^;
- Preparation and application via web of a questionnaire of interest, with information on the profile of the participants, their previous knowledge about concepts related to the research issues as artificial intelligence, human-computer interaction, social networks, etc., participants believes about the phenomenon of death, their interest of being part of the focus group, and their availability; and
- Production of a banner used for promotion via the social network Facebook and WhatsApp in order to seek out available volunteers via questionnaire.

These steps were taken to assemble a group of volunteers with varied points of view on life, death, and computing. The questionnaire was available on the Web for a week. There were no hard inclusion/exclusion criteria, since we wanted a group with as much diversity as possible. The only factors that influenced participation were individual interest in the study and participant availability on the day and time the group was scheduled. Subsequently, the questionnaire respondents were informed, via e-mail, about the time and place of the focus group meeting to start activities. A WhatsApp group was formed with all those interested in the focus group to facilitate communication. The meeting consisted of the following activities:

1. Reception of volunteer participants: when they arrived at the place on the scheduled day and time, they were welcomed and each member received a pen, paper for notes, and the consent form to read and sign
2. A brief introduction to the research topic; and
3. Beginning of the focus group with a question aimed at identifying which members have already had the experience of dealing with death in the digital world and whether they had knowledge about ways or tools for treating it.

Alternately, excerpts from an edited episode of the “Black Mirror” series [13] were reproduced, followed by questions.

Participants were encouraged to discuss what they felt when they saw the scenes, specifically the excerpts from the video belonging to the first episode of the second season “Be Right Back.” The reason for choosing this particular episode is the plot: Ash gets involved in a car accident and dies. Martha, his wife, finds out she is pregnant. And to reconnect again with her deceased husband, Martha decides to participate in a program that uses experimental software that promises to “resurrect” and “immortalize” Ash.

The episode was edited in 3 excerpts:

1. The funeral and the suggestion to immortalize someone recently deceased;
2. Contact with the deceased digitally immortalized via texts and;
3. When the interaction with the digital immortal deepens with an “avatar” of him.

We also conducted mediation of discussions with two neutral interlocutors (members of the research team). Their presence was just to encourage the group to discuss the issues seen in the videos, question the group to elaborate on their opinions, and keep the focus of the discussions. During the activity, the focus group had to answer the following 5 questions:

1. What is your knowledge/living experience about death in the digital world?
2. What are the forms/tools that treat death in the digital world?
3. (After the first video snippet) What would you do if you had to make this decision: to use or not use software to communicate with a known deceased relative?
4. (After the second video snippet) What is your opinion about the possibility of keeping someone alive through technology, for posthumous interaction?
5. (After the third video snippet) What do you think about having a physical manifestation of this recently deceased user via an avatar?

These questions were selected by the first and second authors after thorough discussion and were chosen based on relevance to the study’s purpose, participant’s time commitment and minimizing burden, and maximizing participant engagement. The first two questions were both icebreakers, as means of making the group members more comfortable with each other and more familiar with the topic; they were also to see if participants had experienced (first hand or not) how death was mediated in digital form (e.g., in social media, memorial sites, online funerals, etc) and if they knew about softwares/tools/digital platforms to manage such events. Questions 3, 4, and 5 were related to the video snippets, where we wanted to collect their general opinions of different forms of digital immortality, starting from the inception of the idea and ending on a full-body recreation of the dead.

At the end of the meeting, we conducted a synthesis of the group’s thoughts on the theme and acknowledgments for the cooperation. Subsequently, we inquired for their participation in future meetings to discuss other aspects of the theme.

The preparation of the script, banner, and questionnaire was conducted in the beginning of May 2017. The questionnaire was online during 2 weeks. In the meantime, the first and second authors sought to book one of their institution rooms to perform the focus group activity. With the room booked and the participants who had expressed interest via the online questionnaire, on May 27, 2017, the meeting was carried out and lasted 2 h.

After the activity, audiovisual recordings were transcribed. The analysis and classification adopted in this paper is oriented by Bauer and Gaskell [57]. The analysis was carried out manually, initially consisted of the first author selecting the most interesting statements (opinions, possibilities, positions, statements, stories, experiences, among others) from the transcription of the focus group activity. The second author would also help during the selection/rejection process both by having access to the transcript, the table with the selected quotes, and discussing with the first author over why a quote was kept or not, and, finally, the classification of the statements according to human values as evidenced by the studies of Pereira and Baranauskas [47] with the following script:

- Select the statements that brought significant opinions and concerns about digital immortality;
- Classify the statements according to the values prospected by Pereira and Baranauskas; and
- Analyze whether the values initially indicated for each speech are adequate, identifying the excerpts of the statements that reveal concerns about the values.

The classification of values was performed by the first author of this article and reviewed by the other authors. The next section presents the synthesis of the results and the main points identified from the analysis.

## Findings

The activity with the focus group was successful, promoting the engagement and intense participation of all participants. Two hours of audio recording were performed, which were transcribed into 39 pages. Twenty people were invited and 8 accepted the invitations and participated in the activities on the scheduled day.

In this section, in addition to demographic data, the main issues and questions discussed with the group are presented. To ensure anonymity, participants are identified by the acronym P (participant) followed by a number (1–8).

### Demographic data

From the data collected via online questionnaire of the 8 participants, 5 are men and 3 are women; 3 are between 18 and 25 years old, 3 between 26 and 35 years old, 1 between 36 and 45 years old, and 1 over 45 years old. Three participants are undergraduate students (1 in Computer Science and 2 in Information Systems), 2 are university professors (Law and Pedagogy), 1 is an Information Technology analyst, 1 is a digital media manager graduated in Marketing, and 1 is a high school student.

About Knowledge in Computing and Posthumous HCI, participants were asked about a variety of terms specific to the areas and answered which ones they felt confident to explain. Of these terms, all participants felt confident to explain the Internet concept; 7 would know how to explain social networks, as well as software, hardware, and artificial intelligence; 5 feel comfortable explaining what algorithms are; 4 can explain what neural network, big data, machine learning, chatbot, and digital memorial are. Only 3 participants answered what is digital legacy. Two participants know how to explain posthumous data, and only 1 knows how to explain what posthumous interaction means.

Regarding “Death,” participants were given objective options derived from Maciel’s research [58], in particular about what interviewees’ understanding of “death”: 7 participants agreed the event of death is “natural, inevitable”; 3 participants agree it is “a continuation of divine plans...” as well as “an inexplicable event, a mystery”; 1 participant agreed that death is a “biological failure.” And 1 participant agreed death is “the finite value of carnal life only” while another one agreed it is “a passage into a new dimension of the Spirit after the end of the physical body”.

About knowing the term “Digital Immortality,” before the survey, 5 participants declared they knew the term. All the 8 participants declared an interest in studying the issue of digital immortality in depth. As for religion, 3 participants claimed to be Catholic, 1 Evangelical, 3 no defined religion, and 1 is Wiccan.

### Qualitative analysis

When analyzing and classifying the values raised by the participants, we used the classification scheme proposed by Pereira and Baranauskas [47] to organize them. This scheme was adopted because it offers, as a starting point, a set of values mapped and discussed in software intended to promote social interaction. Furthermore, the scheme allows consideration of technical, personal, and formal issues in an interrelated way, which enables a broader understanding of the identified values.

Table 1 shows the classification process carried out, containing participants’ identification, the transcription^4^ of their answers and comments, and the values identified in the excerpts. Values are indicated in bold inside brackets.

**Table 1:** Examples of the values attribution on the transcripts of participants’ comments and discussions

Transcripts	
**Id**	**Comment**
P1	“I also think (the decision to immortalize or not) should come from the person and not from his/her close relatives [Autonomy] because, for example, if I die today, it is obvious that my family will be influenced by their loss [Relationship, Emotion and Affection]... so if I say ‘I don’t want to’, they will have to respect what I want, do you understand? [Trust, Standards, Rules and Policies].”
P2	“Some days ago I was seeing the Facebook of L., our colleague. She passed away a while ago, 2-3 years I guess... and I see that I was looking at her facebook profile because of our event today in order to see how that works [Object]... people use that search feature... and so they tag her on their facebook posts and photos, and her profile remains active [Identity, Sharing] as if she was still updating her profile. [Privacy, Presence]”
P4	“So, as the subject is new, people didn’t realize that all that personal data was going to stay there online … [Object] when it becomes a memorial it increases the symbol and its mechanics even more [Reputation, Visibility], and it’s a tomb as well, so it’s possible that something will be different … And it’s really strange [Emotion]. And what if whoever passed away never even agreed to this? [Autonomy; Norms, Rules and Policies, Informed Consent] and his or her family decides to keep a memorial [Relationship]... then, there is an open profile and the owner of that profile has no opinion about that (the death of the user who owns the profile)... then you go there and put whatever you want and tag... [Trust] And it is no longer a memorial … it is something else.”
P5	“I am a content producer [Identity, Visibility] too... I write, I make videos, I do some things like... I know this is going to be my legacy, so I think it somehow helps people [Sharing]. This (legacy) will be my memory [Ownership]. My immortality and so on... I don’t know if it will be just my profile there in my memory, as there are other ways to leave (a legacy).”
P6	“Imagine as if today you could have an avatar of Jean-Paul Sartre [Identity], for example, the father of existentialism in modern times, and could having contact with him, going to a French university where his avatar was there [Object], and you could have a class with him... there, with all the data [Availability], an avatar with all his publication, all his academic production in a motherboard and he would be able to answer questions and dialogue … [Usability, Scalability, Adaptability]”
P7	“It is as if we don’t... inducing to immortalize this person... why is it relevant for future generations? [Reciprocity] In contrast, we have the family’s rights after the person has passed away, and we don’t have the right to, let’s say, ask what they want, and we end up missing the point in this discussion [Autonomy, Informed Consent, Norms, Rules and Policies]... why would I like to immortalize? Because it is important for future generations? Or I should give the power to the person’s family to be able to decide this … something that has exactly what has already been said: that sentimental letter from that family bond or that letter of relevance to society [Emotion and Affection]”

After organizing and classifying the comments from participants, we counted the most frequent categories and values to identify the main concerns of users regarding digital immortality. Table 2, part (I), presents the number of comments aggregated into informal (personal), formal (social), and technical levels of formality, while part (II), part (III), and part (IV) of the same table, present the number of comments identified for each value level.

**Table 2:** Number of comments identified for each value level

Value type	Value level	Number
(I) All	Informal	36
	Formal	31
	Technical	9
(II) Informal	Identity	7
	Emotion and affection	6
	Reciprocity	5
	Autonomy	5
	Visibility	5
	Privacy	2
	Trust	2
	Informed consent	2
	Reputation	1
	Presence	1
(III) Formal	Object	10
	Relationship	6
	Property (ownership)	5
	Standards, rules, policies	5
	Sharing	3
	Conversation	2
(IV) Technical	Adaptability	4
	Usability	3
	Scalability	1
	Availability	1

Some values did not appear in the participants’ comments and, therefore, are not listed in the presented tables, such as the formal values of groups and collaboration and the technical values of accessibility, awareness, appearance (esthetics), portability, and security. On the one hand, not all values should necessarily emerge from participants’ comments as the focus group is not an exhaustive technique. On the other hand, their absence suggests that these values did not appear as a priority for the participants during their conversation. Problems with values are usually identified when technologies are already in use. Therefore, their absence in the discussion may indicate issues that would only be identified when people are effectively using digital immortality resources and face problems with them (e.g., accessibility, awareness).

The results reveal a strong concern with values of informal and formal levels, and little attention was given to values of technical level. This was expected because the focus group was not a predominantly technical discussion, but a session about the social role of technologies, their use, and their impact on people’s lives and continuously after their life. However, because among the participants there were professionals working in the Information Technology (IT) area, we were expecting more discussions related to technical values, such as accessibility, awareness and security.

Participants who lacked specific knowledge in computing (P1, P2, P6, P5, P8) showed a greater concern with informal values, followed by formal values and almost no discussion on technical values. In turn, the IT subgroup (P4, P3, P7) ended up having more balance in the discussion between values of all levels.

Participants’ religion was not a critical factor about what values the group decided to address. Despite the initial impact with the theme, gradually, during the activity, participants became more comfortable to discuss and express their opinions, talking to each other and engaging in discussions. Something similar was reported by Galvão and Maciel [37], where authors highlight that as discussions were evolving, participants started understanding their possibilities to express themselves, discussing, sharing their ideas, making their opinions more flexible, at the same time they were aware of the challenges and limits of this theme. Religious participants, such as P6 and P7, demonstrated possibilities in applications for digital immortality in education and as a tool to help dealing with the pain of grief.

The most addressed informal values were Identity: the individual’s “self”; the expression of elements of a person’s personality and individuality; Emotion and Affection: feelings of an individual such as well-being, pleasure, fun, tranquility, involvement, annoyance, disappointment; and Reciprocity: feeling of reward or mutual benefit with performing a task, using or applying some effort on something.

The concern for these values by the participants stems from how they imagine that loved ones would deal with digital immortality and also in the opposite roles where they were being digitally immortalized by their loved ones. A similar concern that Khalid and Dix [59] found out in their focus group: “(...) Participants are generally aware and attentive to their audience and their reaction (...)”. As evidenced by P7 in our study: “It is as if we don’t... inducing to immortalize this person... Why is it relevant for future generations? In contrast, we have the family’s rights after the person has passed away, and we don’t have the right to, let’s say, ask what they want, and we end up missing the point in this discussion.” Participants’ fears about the immortalization of their loved ones are evident, especially without their consent. This can be partially explained by the very informal nature of the manifested values: informal is related to everyday aspects of life, to the way we relate to each other, live, understand, and approach life. The informal is tied to cultural and personal aspects of life in such strong forms that sometimes they are difficult to be understood, grasped, and explained, usually arising when something goes wrong. Therefore, it is expected that informal values reveal participants beliefs and fears about what they live, expect, think and feel.

Participants exposed similar views on the importance of informal values in a hypothetical digital immortalization software. Participants stressed they feel more comfortable when they can control and make their own decisions, as stated by P1: “I think it (the decision to immortalize or not) should come from the person and not from close relatives.”. Then, participants prefer to keep to themselves the power to decide whether to immortalize (or not) their loved ones, allying such a decision to their personal values regarding the manner of managing posthumous digital life in accordance to what they consider appropriate to their needs. This result communicates a strong emphasis on autonomy as an informal value, extending its importance and reach beyond life.

When it comes to values manifested at the formal level, the Object—an artifact around which social interactions occur and are developed [47]—was the most discussed value around which participants developed discussions and exposed their concerns. For instance, P5 wondered about the different objects that could characterize his/her legacy: “I’m a content producer... I write, make videos and stuff like that. I know this is going to be my legacy(...).” Other objects cited by participants were related to personal data and posts in social networks (P4), pictures and thoughts (P7), and books and intellectual work (P6).

Another value strongly discussed was Relationship—some type of connection, social bond, between two or more individuals [47]. For instance, when discussing the interaction with digital memories or representations of their loved ones, participants raised concerns regarding changes in their relationships. When P7 exposed that “it would be interesting to have a tool where I could transmit my thoughts to the loved one, (...) transmit something I would like to pass on to her and get a digital response back.”, P4 raised concern on the different types of relationships: “What makes them different is much more like an emotional bond, like a bond with a father, uncle, etc., and you give meaning to this bond... but the bond you have with another person can be different, it’s a different relationship, and that relationship will change completely whether you’re going to open it or not [in a system]”.

Other two values also received strong focus: Ownership (Possession), as the right of possession over an object or information and the actions that can be performed on that object [47], and Norms, Rules, and Policies as aspects that govern, regulate, and determine how the individuals behave, think, make judgments, and perceive the world. P6 gave examples of famous authors (in Law) whose families did not negotiate copyrights with publishers, making their work fall into disuse, while other authors whose families granted copyright continued to be cited, studied, and republished, some having their works transformed and updated by discussions and future work of their disciples. For P1, people have rights over their objects and these rights must be respected: “Particularly I wouldn’t leave anything of mine to my family. (...) So, if I say that I don’t want, they will have to respect what I want (...)”. However, as P4 suggests, people are unable to know in advance all the possible use of their objects in the future: “as the subject is new, people didn’t think that all that personal data was going to stay there (...) What if the deceased has never agreed with this?”.

As the previous examples suggest, values in the formal level tend to reveal aspects of people relations and regulations as a social group, usually depending on social and cultural norms shared by a group of people.

Participants also questioned and raised discussions on how a digital immortality software would work. For example, P4 during the discussion: “The program starts to learn new things and... And it has a self-awareness (consciousness) that it is an AI, (...) and that changes how it behaves, so it has a concern about itself and knows the path it uses (to interact)”. For P5, “... the more the software interacts, the more it learns how this relationship will be, and the more real it gets.” These sentences are examples of what human interactions can happen in between the present and the past in an innovative way, as suggested by Bell and Gray [8]. These statements exemplify that digital immortality, when applied in different contexts, can provide innovative interactions that extend the possibilities of digital immortality beyond a simple digital memorial.

Promising possibilities for digital immortality were detected by the participants, as mentioned by P6 when the participant envisaged an educational tool: “Imagine as if today you could have an avatar of Jean-Paul Sartre (...), going to a French university where his avatar was there, and you could have a class with him....” And also by P7 when discussing digital immortality as a mechanism to help people to deal with the pain of grief: “It would be interesting to have a tool that I can be able to transmit my thoughts to the loved one...”.

For values at the technical level, the most addressed values were Usability, referring to consistent, controllable, and predictable interfaces [47], and Adaptability, referring to the possibility of changing an application according to its context of use. Scalability as the ability to support a growing number of users and to handle an increasing amount of information [47] and Availability as the possibility to have access to resources anytime, anywhere for anyone, were also mentioned. Values in the technical level tend to reveal the importance people give to technical qualities or the expectancy they have regarding ideal rules that should characterize or regulate the technical system.

According to P5: “So... probably, the more it interacts, the more the software learns how this relationship is going to be, and the more real it gets...”. And P4: “(...) it seems that there is a mixture of memories with a program that is prepared for whatever... Facing countless events (...)”. Participants, when discussing problems that a software that promotes immortality may face, agree the software must be flexible, easy to use, and not restricted to a single perspective regarding the way digital immortality may be applied. It must respond adequately to the different situations it faces and must provide users the necessary resource for them to understand the software capabilities and limits while using it.

From participants’ responses regarding digital immortality, we can see informal and formal values were a priority, permeating their discussions. Until we have systems that replicate digital immortality similar to the way fiction is already doing (e.g., Black Mirror series [42]), participants have expressed concern about how their loved ones would be represented in digital immortality, especially regarding the emotional side and the consequences that digital immortality can have on relationships with the living and the person digitally immortalized. In such concerns, we can see values of the three levels interacting to each other: while informal values are communicating participants’ concerns regarding their relationships, feelings, social consequences, and so on, formal values appear as both the need to regulate or decide what is right or not, and the need to develop a shared understanding of basic issues and accepted behavioral patterns regarding a topic that is still new but already present in our lives. Technical values, in turn, appear mainly as desired characteristics and quality attributes of technology to support digital immortality and people who exist on it, in its broadest sense.

Discussions revealed participants perceive digital immortality as a way to keep loved ones virtually present for future interaction, a way to deal with grieving pain, to keep the memory of deceased loved ones alive, and also a new way to learn from the data of deceased people interactively. All participants agreed that, in order to use digital immortality systems, it is necessary the system to be flexible, capable of changing over time, respect the memory of deceased ones, allow interactions between the virtualized dead and the living, and especially that it aligns and respect for human values, both of its virtualized entities and its users.

The results also revealed values that permeated the discussion about digital immortality, its characteristics and implications. Digital immortality requires that the understanding of interaction in the dimensions of time and space to be revisited beyond the individual’s life, opening space for considering other forms of interaction continued in time and distributed in space. Understanding digital immortality as a value will be necessary to understand how to develop technological solutions suitable for human life in its different cultural contexts.

In this sense, regarding the set of values used as a basis for the analysis of the participants’ comments and discussions, we highlight the set is comprehensive but not exhaustive. As Pereira and Baranauskas [47] point out, using a set of values as a starting point is useful, but one must remain open to values that may emerge from the context of analysis. Several moments and words from the focus groups suggest important concepts and concerns that may be understood as values being communicated by the participants. The very question of digital immortality itself can be understood and discussed as a human value that re-signifies our understanding of the basic dimensions of time, space, and interaction, extending them and giving them different meanings and dimensions we are used to. For Hall [15], interacting is to be alive, and failing to interact represents death. However, discussing digital immortality as a value centered on interaction requires us to develop an even deeper discussion where interacting means existing, being, in different ways and forms.

**Fig. 5:**
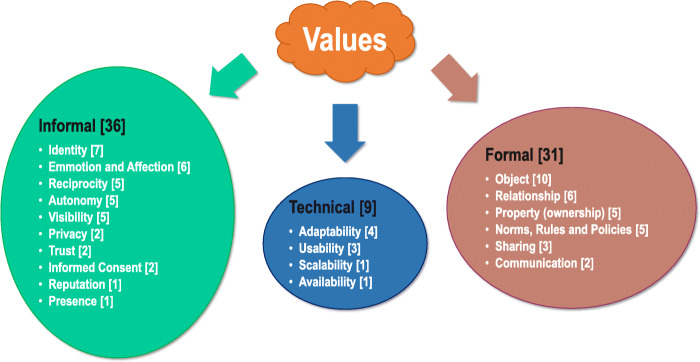
Values identified from participants’ discussion

Figure 5 summarizes the values identified in the focus group analysis. As results achieved with this focus group are successful, it is worth replicating the focus group in other sessions and comparing results with other participants in different contexts to better explore the wider aspects of the study topic such as discussing the same questions and video clips with a national/international focus group, to have participants with a wider age difference between them, and to compose a group (or subgroup) of participants who are IT professionals in order to discuss more technical aspects surrounding digital immortality. Such distinct groups could shed light in aspects that different demographics prioritize and allow for the comparison of results between them. Another important point is to guarantee as much of an unbiased approach as possible towards the topic in order to better understand the reaction of the participants and the benefits of the theme, by using a better selection method for the quotes involving more discussion with a more diverse set of researchers, as well as statistical analysis of the quotes in order to identify feelings, patterns, and better explore the volume of data collected with a bigger sample size, something that was not possible to achieve widely in this study.

### Limitations

As for the limitations of this study, the focus group is small (8 participants). Although it has enough participants for a qualitative study, each with differences in ages, personal experiences, professions, religions, etc., the group has a relatively similar cultural background, as many are residents of the same city and share similar daily life problems, routines, etc. Most volunteers were relatively young, in higher-education and digitally literate, so it was not possible to observe reactions and thoughts of people who might have been significantly less skilled or knowledgeable about digital immortality. The group was only dedicated to discussing technology for digital immortality, not to use and experience it, favoring prospective and general discussions over analytic and specific ones. Therefore, this study does not allow us to claim for generalizations but, instead, provides us indicators of how users understand, envisage, and worry about technologies to promote digital immortality, helping us to prospect and anticipate issues that must be considered when designing and evaluating them. Also, not having an additional separate focus group in order to perform the same methodology and compare results does not allow for an understanding of a wider variability of our findings.

### Notable highlights

Aside from notable quotes analyzed through a value perspective, during the focus group activity, there were discussions and opinions that while did fit any of Pereira and Baranauskas [47] classifications, they pointed out to important considerations and discussions regarding digital immortality. While they are not the focus of this study, we will briefly discuss them here.

Quote 1—P4: “(...) how does it (the social media profile) transforms into a memorial and the person, (who passed away), he/she has not decided about it. I think that really is a privacy invasion regarding the memory of the (dead) person, if you go look at their (social media) profiles various times or I don’t know. Or the own family to do it, sometimes they think that they are reworking something, but depending on the situation, because to me what is worth is the opinion of the deceased”

While there are clearly concerns about norms and privacy exhibited by P4, there is also another point, albeit not very well expressed by P4 that does need attention: before we focus on digital immortality, is it not necessary first to have solid legislation about what to do with digital assets? While in this scenario, its easy to be compelled to simply obey whatever the deceased wishes. However, what if the deceased has not declared what should be done with his/her digital assets before passing away? Should the family be responsible for it and solve the issue themselves? Should only the party responsible for hosting the files have access to the deceased digital assets? Should the deceased’s data be donated to public libraries or universities?

The fact that we do not have guides or an idea of how to adequately approach this question and yet there are already a wide variety of software that are suited to posthumous data preservation and digital immortality [60] is quite an example of “doing it first, thinking about the conversations and consequences later.” While this issue in particular is out the scope of this study, this lack of formal and specific legislation on the topic or mainstream discussions about what to do with the data of the dead [24, 25] still generates some frustrations. The companies who host such content are not always willing to share the data they collect even if it is for an individual who passed away, lawmakers do not have posthumous data as a priority, and, to the general public, it is not a widespread issue yet. This is a matter that will require both careful studies and ways to encourage wide public discussions.

Quote 2—P1: “(...) I remember to this day this saying of my grandfather, my grandfather after his first cardiac arrest he said: “I know I will die, each one of you will take care of your own lives, and don’t need to stay here putting up with me, it is a fact that I will die, I will be buried, and everyone will move on with their life, there’s no need to stay there (near the body)”.”

P1’s grandfathers line in particular is quite important; the death of a loved one is painful both for the living and the soon-to-be deceased. Even in this moment, P1’s grandfather hopes that family members will move on with their lives. Digital immortality’s focus is mainly on the preservation of human data in an interactive format for an indeterminate time frame. Digital immortality in general somewhat an antithesis to P1’s quote and a concern raised by Gulotta, Kelliher, and Forlizzi [61]: would future generations even want to go back and remember the dead after so long?

The desire to be remembered is a very human trait. As aforementioned, people achieve immortality via their works and bonds they form with others throughout their lives [34]. And on the other hand, the desire to be forgotten and to have your digital assets deleted is relatively easy to configure and often a option in most services [5]. But for those who want to be remembered, how to help them? In Gulotta, Kelliher, and Forlizzi [61] studies, they point out an inevitability surrounding the preservation digital legacies: future generations may not perceive or understand them the same way as the creator or people from the same generation as the creator. The authors suggest that in this case it is necessary for softwares to focus more on the process instead of the results. That is to say, immortality is not an exact result, and its meaning will differ greatly from individual to individual. Therefore, it is better to guide users on how they mold their digital immortality via key questions for them to better present the narrative of their lives (i.e., how would you like people to perceive your life’s story? What would be the most important aspects of your life you do not want to be forgotten? Would someone, outside your family and friends, understand what kind of person you were? etc.) Considering the possibilities, what if someone wants to share their digital data, legacy, and immortality, but does not want to be identified? Is it necessary to create a pseudonym and generic avatar? Who would receive such data? What legal procedures and regulations are necessary in order to make this possible while reducing the possibility for abuses and loopholes? Or what about someone who does not want to be digitally resurrected?

Perhaps a focus shift on how digital immortality is approached in general is necessary. Instead of treating it as an objective goal, to take it as a process may be more productive, in order to both allow developers to design software that informs users to achieve their posthumous goals and for users to personalize finer aspects of digital immortality that are not always expected or predictable by the developer.

Quote 3—P5: “Now there’s an interesting parallel: Organ Donation. Yeah... Even though you may want to donate, your family will have the final say, (unless) you had left (a manifestation of) explicit desire (...)”

P5 raised an interesting point: What if we were able to give or donate our data similarly to an organ donation process after we pass away? Some authors propose that the possibility to study, for an example, a historical event via people’s comments instead of the news could present a valuable and unique way to interpret human history [32, 62]. Especially now, in the information age, where we are producing extremely high volumes of information at an unprecedented speed. It is not hard to see that there is some merit to P5’s idea.

Some refinement and long careful studies are needed. What kinds of information can be donated? Should the data donated be thoroughly edited in order to protect the identity of the deceased? Who to donate this data to? Questions like these are simple to make, yet very tough to answer. The data people produce on a daily basis is not only complex to grasp but also widely variable both in nature and also on where it was produced. Which not only becomes hard to separate what is meaningful enough to donate, but also the varied nature of digital services, each one with their own terms of service, based on one or several countries legal systems, as well as the corporate culture each service is established upon; makes it a very layered issue, which it may be impossible to have all involved parties to completely or partially agree to giving data away for the sake of a single user [9].

Also, unlike organs, data does not have an expiration date, it does not posses the same urgency; due to this, it is possible a single donation process may end up in legal limbo. While there is software that allows the user to delegate their data to digital inheritors, there’s a very prominent need for contingency measures and systems for digital preservation [60] (and by extension digital immortality), so that in case the deceased’s data, if not handled by the family, or without explicit desire by expressed by the dead (be it for its deletion, donation, or inheritance), could still be of some use in future by scholars, historians, researchers, and common people as part of a public data domain of sorts. It is interesting to consider co-design and participation of both users and developers in this process. This would be important to ensure an agile development environment, granular controls in what can be shared and what should be kept private. It is also interesting to consider what it would look like to educate communities related to their post-mortem legacies, digital data rights, and their digital immortality.

## Final considerations

Our study, based on focus group interviews, revealed a set of human values that are often not considered concerning digital immortality. The study also identified the need to consider immortality itself as a human value to be considered. Unexpectedly, for being a morbid theme, participants demonstrated interest on the topic since the technology has made it possible. They foresee the applicability of digital immortality in other important areas and contexts.

The participants declared to perceive digital immortality as a way to keep the loved ones virtually around for future interaction. They also find it as an interesting opportunity to deal with grieving pain, to keep alive the memory of the deceased loved ones, and also to learn more of the deceased ones from interacting with their stored data. All participants agree that digital immortality systems must be flexible, capable of changing over time, and allow communication between the virtual dead and the living. They also agree that such systems must honor the memory of deceased ones, respecting the human values, both for the virtual entities and for the users.

Participants demonstrated a clear concern related to digital immortality, especially towards who will use it and how it will be used, as suggested by the existential themes for the HCI community. They raised questions for the future of society with digital immortality such as the meaning the digital artifact takes for future generations, the benefits it could bring, and the risks and the ability to mitigate possible harm from it. These questions put into perspective how much someone can be transformed by the experience of digital immortality, both for the immortalized deceased and for the users interacting with this technology. While it is not yet possible to understand all the impacts that digital immortalization will bring, this study allowed us to observe how important systems that deal with sensitive subjects, such as death, must consider human values in their development. The system should be open to allow changes that better respect the plurality in terms of cultural, religious, social, moral, and other aspects that define mankind.

The participants’ opinions indicate that digital immortality systems should be prepared for future possibilities of interaction that respect the following characteristics:

- The object: the form from which the digital immortality system will take place must allow interactions that respect the relationship of users with their loved ones;
- The expression of values, such as emotion and affection: the feelings that users may be dealing with related to death; and
- The identity: since both, users and the deceased, share memories of their relationship. In order to preserve this data, the system must have norms, rules, and policies that protect and assures these virtual properties are neither improperly used nor controlled by other users or malicious third parties.

The system designers must consider the issue of reciprocity. Users want to feel that the effort of immortalization is not being in vain and that it benefits other users while respecting the memories of the deceased. Also, the system must be adaptable and capable of dealing with the scalability of the problems it may face, even in situations that may not be completely foreseen in its original design. Finally, the system usability must be considered for easy to understand and to allow users to make decisions aligned with their values.

Software systems have taken on such an active role in our contemporary lives that they are also shaping ideologies and cultures, extending their purpose beyond mere tools, as suggested by Balkin [31]. Our study allowed us to observe the importance of considering the human values in the development of software for digital immortality, showing how users perceive digital immortality both positively and negatively through the values the software can evoke.

Through our work, it is possible to understand that for the development of digital immortality systems need to balance complex technical and human factors such as:

- Users’ personal history with the deceased and how systems can help preserve it.
- Ways for posthumous systems to be customizable and respectful to users’ technical and cultural needs (e.g., how and where do they want this data to be stored, ways for this data to be shared safely, and how can their funeral rites transition as smoothly as possible from the physical space to the digital space).
- Whatever form digital immortality may take, (a memorial, digital copy, robotic avatar, and many others), it needs to be consistent with its design goals and also the previously presented points.

While these factors are a broad rough sketch, it is certain that as technological innovations become more integrated with our personal lives, considerations with values and interdisciplinary studies in computing system design are a must in order to help software and hardware to better assist people in dealing with a wide range of sensitive issues.

Despite the limitations of the environment in which the study was conducted, digital immortality appeared to be a promising alternative for the treatment of posthumous data. However, for digital immortality to reach its full potential, in a way that respects human sensitivities at the time of death, the discussion should go beyond the human-computer interaction issues abridging other areas of knowledge to consider the needs of users, family and loved ones. This research is part of the Data Beyond Life (DAVI)^5^ project, which seeks to carry out works to discuss issues related to the post-death digital legacy issues.

As future works, due to the interdisciplinary nature of the theme, it is worth understanding the population’s interest in the technology to build digital immortality systems as a way of dealing with the pain of death. Our study left open some interesting questions, worth an investigation, such as the following: how much suffering with the loss of something should be interpreted as something bad? Would the creation of systems designed to deal with death foster a society that does not accept frustration? How can these systems respect the wishes of the dead and the needs of the living? and other similar issues.

Alongside these questions there are important dilemmas surrounding digital immortality that need careful and critical exploration. For example, what about considersations for a public immortality vs a private one and the possible pros and cons of each. How does an individual’s social status influence their digital immortality (e.g., where are their differences between digital legacies of famous vs unfamous people)? Is digital immortality a need or a luxury? Where is the boundary between this new digital immortality space and existing immortalities, since some individuals are already immortal due to their works and deeds? Who, besides tech companies (which could profit for providing such service,) would need to build or use digital immortality? Finally, where does value reside—is immortality a value or is it mortality? Or more so, are these both constructs with associated events or lack thereof (i.e., death) around which we have human values pertaining to self-continuity, integrity, privacy, autonomy, and control?

As aforementioned, there are many complex variables to digital immortality, each belonging to a wide set and subset of layered issues that may need its own dedicated research agenda to be discussed in an adequate manner. A critical examination of digital immortality is crucial in order to access its full potential and to direct it as resource to aid the difficult times and questions brought by the death of loved ones. While the initial focus of this paper was within a small sample size from Brazil, future works will need to explore considerations and values on a larger level, since digital legacy and digital immortality questions are intimately linked with our ever-growing, increasingly integrated society.

Discussing digital immortality from the human values’ perspective is useful for making us reflect on socio-cultural issues that go beyond the technological challenge of building the systems. Of course, this is not the only way to discuss the topic; however, our study shed some light on human aspects that have been neglected and covered by the technological challenge of creating the means (the software). Death is a fate and a delicate issue to be handled. Digital immortality might be feasible but requires dealing with a myriad of issues in which human values are a must.

## Data Availability

The anonymity of the data collected, used, and analyzed during the current study are available from the corresponding author on reasonable request.

## Footnotes

1 occurring after death - Merriam Webster dictionary

2 This paper is an extended and revised version of the paper [28]

3 Ethical Committee approval under number CAAE n ∘:64403416.6.0000.5690

4 Transcriptions presented in this paper were translated from Portuguese to English by the authors

5 Project DAVI - https://lavi.ic.ufmt.br/davi/
